# The changing role of marketing: transformed propositions, processes and partnerships

**DOI:** 10.1007/s13162-020-00179-4

**Published:** 2020-10-26

**Authors:** Kaj Storbacka, Ted Moser

**Affiliations:** 1 Hanken School of Economics, Helsinki, Finland; 2 Prophet, San Francisco, USA

## Digital transformation of marketing

Digitalization affects every aspect of a firm’s business model–from front-end to back-office, from how firms create value for their customers to how they capture value– and doing so can reshape every facet of the firm. By adapting their business models to the possibilities of technology, firms are facing an accelerating transformation of their activities, offering new opportunities for “out-of-the-box” development of new processes and tools, which effectively challenge deeply engrained functional silo-based thinking.

Despite the ubiquity of digital transformation, much academic research still seems to take a functional view (Verhoef et al. 2019), where information systems look into the development and adoption of specific technologies (Nambisan et al. 2017) or analytics schemes (Davenport and Ronanki 2018), strategic management research focuses on understanding the role of new digitalized business models (Foss and Saebi 2017), and marketing research focuses on what is generally called “digital marketing” or the development of an omni-channel environment (Verhoef et al. 2015; Lamberton and Stephen 2016; Kannan and Li 2017).

Lately, some researchers have argued for multi-, cross-, and/or trans-disciplinary approaches to digital transformation (Kumar 2018; Crittenden and Peterson 2019; Verhoef et al. 2019; Grewal et al. 2020). Instead of digitalizing existing practices within silos, we need new ways to cross-fertilize the insights from different functional development streams into a coherent firm-wide approach. Staying in the functional silos may, for instance, lead to ignoring relevant opportunities for new work divisions between the functions.

This view of digital transformation challenges many of the fundamental theories and concepts that management and marketing have been built on. This drives us to evaluate, for instance, how economies of scale work, if transaction costs can be used to define firm boundaries, how we define markets, and how to measure value (Storbacka 2018). Consequently, marketing scholarship actively questions the boundaries of marketing and calls have been made for the need to engage in research that actively removes the blinders of the “mainstream assumptions, theories and methods” (Moorman et al. 2019, p.1), with the aim to uncover new ways to look at value creation in the market.

Practitioners are facing this dilemma in real time. This interview with Ted Moser, who advises firms on the digital transformation of marketing, aims at identifying “boundary-breaking” ideas that can form the basis for new types of research questions.

Ted holds an MBA in marketing and strategy from the Wharton Business School. He has 30 years of experience advising leading firms on growth strategies that cascade from business model innovation to go-to-market transformation. His work spans markets in digital, medical, and industrial technology, plus financial services and media entertainment. Ted has lived and worked in the US, Europe, and Latin America, and has worked extensively in Asia.

Ted is a senior partner in the San Francisco office of Prophet, a strategic brand and marketing consultancy with offices around the world. Prophet helps leading companies digitally shift their innovation, marketing, and branding to achieve transformative growth. Ted began his consulting career at Corporate Decisions, a growth strategy specialist spun off from Bain and Co. and led their European practice. After Corporate Decisions was sold to Mercer Management Consulting, Ted led Mercer’s US West Coast practice and chaired its global service offer innovation committee prior to joining Prophet.

Ted has contributed to several books on business model innovation, for instance, Profit Patterns, which he co-authored with Adrian Slywotzky (Slywotzky et al. 1999). Presently he is writing a book about the transformation of marketing, rooted in the ripple effects of digital innovation, and this is the focus of our discussion.

## Interview with Ted Moser

**Storbacka**: When I talk to marketing practitioners, they are very focused on digital marketing. However, it seems that you are taking a broader view on how digitalization affects marketing–a view that takes us way beyond digital marketing?

**Moser**: The digital transformation of marketing includes digital marketing, but it reshapes much more, encompassing the “digital ripple effect”. If we examine the question of how digital is changing the role of marketing and the ways marketing is getting done, we end up with a transformation agenda that far exceeds the normally accepted definitions of digital marketing.

To illustrate, let’s start with the business innovations that marketing is asked to support. When a marketing leadership team plans for the next one to three years, the first question asked should be: “How is our firm’s business portfolio changing in search of higher value, and how can marketing support the success of that change?” Most often, digital is changing what the business is trying to do. Firms may be developing new business models or offers that are altered fundamentally by digital. Consequently, marketing is likely to find itself ***marketing things that it never marketed before***. That’s just one example of how digital has a ripple effect on marketing, without touching what we typically call digital marketing.

Another example is the way ***customer expectations and behaviors*** are evolving due to digital innovation. Marketing leaders need to re-assess more frequently how buyers are evolving the way they gather information, come to decisions, prioritize benefits, and experience value throughout the customer journey. The digital ripple effect means marketing needs new and better customer evolution sensing systems.

In parallel, digital is ***creating an explosion of new channels and related new tactics***. We take for granted the existence of websites, mobile sites, and branded apps but these are all innovations in the last 20 years. On the rise now are marketing through a digital assistant, an AR/VR application, geolocation beacon data, IOT streaming data dashboards, and in-product AI chat. All this means that marketing teams need to learn to use new channels that they’ve never used before.

Most fundamentally, thanks to digital the whole ***scope of marketing is changing***. The touch points that marketing has with the buyer are clearly becoming digital and, in that context, we do find digital marketing. However, I would argue that even this is not marketing with the same borders that it used to have. What we call ***digital marketing is increasingly becoming digital selling*.**

What is selling? It’s a one-to-one relationship between a vendor and a customer. A relationship where the two sides are getting to know each other, and as they do, the vendor is reading the clues that the customer provides, to provide a personalized message, personalized advice, and a personalized value proposition. That’s the classic role of a sales rep–to establish a one-on-one advisory relationship that provides contextualized value. So, digital changes marketing by turning marketers into virtual early-stage sellers far upstream in the funnel. Going forward marketing will create that one-on-one relationship with the buyer long before the sales rep does downstream.

This matters because in some product categories, with some customer segments, marketing can keep selling virtually all the way through the point-of-sale. Buyers may no longer see value in speaking to a human being before they choose. At that point, marketing is encroaching not only on selling but on e-commerce and sometimes customer success. It’s not simply taking the dynamics of what we’ve traditionally called marketing and turning that digital.

All of this means that the ***role that marketing can play in a company’s success is changing***. It can ***go beyond creating reputation for the company, to creating relationships and even revenue****.* Early adopters of digital approaches were only on the hook for impressions – mimicking a traditional marketing role - but they’re increasingly on the hook for engagement and leads and going forward may be asked for quarterly revenues and a lower total cost of sales-to-revenue ratio.

Over time the ***cultural ethos of marketing*** will shift beyond creating a digital version of today’s marketing culture. It will evolve toward a culture of digital dialog with customers instead of one-way communication to them, plus a culture of results-generation for the company. With greater visibility into marketing’s impact on the customer, resources will be allocated toward these relatively new capabilities. Marketing leadership’s challenge will be to help a new ethos rise, while still nurturing marketing’s spirit of creative experiential communications.

The final consequence of these changes is the need to redefine the ***kind of talent marketing needs to attract, recruit and keep***. Not only those who understand digital marketing technically, but those who can thrive in the type of environment that the digital ripple effect creates.

I hope this illustrates how profound of a transformation is coming to marketing, one that includes but also far surpasses what we call digital marketing, spanning the entire digital effect.

**Storbacka:** This is obviously the reason why you call for the transformation of marketing. My understanding is that you have divided the transformation into three different types. Could give an overview?

**Fig. 1:**
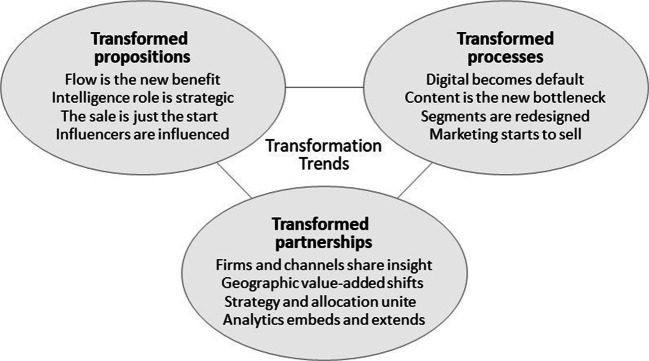
Transformation of Marketing

**Moser:** Yes, let’s discuss three types of transformations: transformed propositions, transformed processes and transformed partnerships. In each of these three areas I will highlight four major trends, so a dozen trends in total. I hope your readers find at least some of these worthy of future research. (See Fig. 1)

## Transformed propositions

Thanks to the nature of digital offer innovation, marketing leaders will need to enable their product and solution marketing teams to excel at an evolved playbook. How to integrate improved flow–workflows in a B2B context and lifeflows in a B2C context–into value propositions as a new type of benefit layered over traditional benefit categories? How to become known as the critical intelligence within the flow? How to market past the point of sale to encourage the customer behavioral change needed to experience the value of new flow throughout the usage lifecycle? How to win over trusted influencers who will back up the company’s value proposition claims?

Let’s discuss these in more detail.

### Flow is the new benefit

In a digital world, ***the innovations to be marketed are increasingly systems that promise new benefits of flow.*** In business markets we call these better *workflows*. In consumer markets we might call them better *lifeflows*. Digital innovations carry the potential for profiling and quantifying the before-and-after value of flow improvement. To do so will require a new level of customer centricity and insight.

What helps products and services to become flow-enabling systems is three-fold. First, sensor-rich offerings–whether a cell phone app, a connected product, an IP-addressed sensing component, or a visible cloud-hosted keystroke–are connecting the product/service to the customer as the customer uses that product/service in their daily lives, generating key data needed to profile their broader activity flow.

Second, programmable software is controlling what was once considered hardware functionality–and can further log detailed product use. This software-controls-hardware logic will continue to expand over time, including extension to smart materials. To support lifecycle usage flexibility and customer data insight, physical product lines are also being transformed into a one “base” with many “bits,” whether those are called razors and blades, coffee makers and pods, power tool platforms and end pieces, even car chassis and replaceable outer shells.

Third, the customer’s use of digital payment methods (credit and loyalty cards, smart phones) is creating a forensic history of economic activity.

***Innovations will be most effectively marketed in the context of B2B customer workflow and B2C customer lifeflow benefits.*** Yet flow-improvements are not something that most marketing teams are accustomed to bringing out. Marketing teams are instead used to promoting static product and solution benefits. To market flow benefits effectively, product and solution marketing will need to much better understand how the customer uses what it is they have purchased. The new responsibility that will fall on marketing is wide-reaching.

We see flow benefit communications emerging. I’ll share two widely recognized examples. When Starbucks markets coffee to me it’s no longer just about great beans and flavors; it’s about the lifeflow of ordering on the smart phone as I’m leaving home and picking up with no wait and no pay as I continue to work. The genius of Apple advertising has been to convert what used to be product ads into 60 seconds consumer lifeflow movies based on what its products enable. Flow benefits don’t replace product or service benefits; they can add a new and often differentiated layer of benefits on top.

In B2B one might think of Total Workflow Benefits as a modern successor to ‘Total Cost of Ownership’. It’s how a new system enables better flow that compresses time, improves customer productivity, and enables innovative outcomes. Similarly, when marketing to a consumer, it’s how the new system enables better flow that saves time, makes loved ones happier, creates amazing experiences, or helps advance a career.

Companies who don’t make or control an entire system can make the mistake of thinking they’re not part of a flow. In fact, they are. They’re just part of a multi-brand system that the customer has stitched together. In this situation, marketing should be advocating the creation and curation of virtual system options through partnerships and APIs. Flow marketing principles will then increasingly apply.

### Intelligence role is strategic

Somewhere in each work–and lifeflow there is, or there will be, intelligent decision-making that adds particularly high value to the customer, brought about by software code and aided by machine learning (ML) and artificial intelligence (AI). As flow benefits are marketed, different companies may claim to be the brains behind that intelligent flow. This claim matters because ***flow intelligence will emerge over time as a customer priority and a competitive differentiator.*** In parallel, intelligence is becoming a powerful new brand attribute that distinguishes modern brands from old ones.

Beyond learning to market flow, it is additionally strategic for marketing to decide whether and how the company can credibly claim that it provides the brainpower within the flow. ***Marketers should aim to win credit for valuable flow intelligence and extend that credit strategically through brand architecture***. Competition will be fierce–both direct and substitute competitors will make claims. The winner of this contest will differentiate today and will hold the high ground heading into a wave of business innovation that lies still further ahead of us–autonomous business.

This trend raises questions around an intelligence brand. On the one hand a dedicated intelligence brand can focus the spotlight on a company’s intelligence value added. On the other hand, it can deposition the master brand. By focusing so intently on how intelligent Watson is, IBM may have made its flagship brand seem less so. Microsoft made different choices, distributing certain types of intelligence responsibilities across several product, platform, and master brands. A key question for all marketers to wrestle with is: “How do we differentiate on intelligence while ensuring that intelligence equities enhance the brands we care about most?”

The branding of intelligence is a deep topic in its own right, and one that we won’t do justice to in this interview. One illustrative example, tied to designing for branded intelligence: it’s best to enable the brand’s value to visually be performed through algorithmic action and expression, rather than just be represented through static symbols. This can reveal the intelligence brand’s essential value, psychology, and ethics.

### The sale is just the start

Customers will purchase digital innovations with best intentions of achieving new flow benefits. Yet it’s not guaranteed that their experience will be positive; they may or may not succeed. In a pre-digital world, the customer’s experience of quality was primarily dependent on product or service performance. ***In a flow world, a great customer benefit experience depends meaningfully on the customer’s own behavioral change–a blend of user engagement and know-how–making lifecycle marketing critical.*** That’s why marketing needs to view the sale as the starting line, not the finish line, for the company’s value proposition.

The implication: ***marketing should allocate resources to enable customer flow success at key points throughout the usage lifecycle.*** The achievement of customer success will do more than raise customer satisfaction scores; it will lead to accelerated growth. Many innovations will be priced as subscriptions or variable utilities vs. once-and-done purchases, putting the customer in a mindset of expecting ongoing value delivery experiences in return for ongoing payment. In this context customer success will raise retention. Going further, lifecycle marketing will enable the company to achieve a trusted understanding of the objectives driving customer usage, leading to improved customer cross-sell, upsell, and advocacy.

As more becomes trackable, customer activities and experiences throughout the usage lifecycle will become more visible–given customer consent, of course. Transformed marketing in a data privacy era will work hard to gain customer consent to data visibility because it is key to creating a win-win lifecycle customer relationship. Companies are now designing services that offer the customer valuable services in exchange for data access. It will fall on modern marketing to support the design, management, and/or promotion of these value-for-data exchanges.

This activity may require marketing to collaborate with other lifecycle programs that go by a variety of names: customer experience, customer success, customer engagement, customer loyalty, renewal marketing, customer marketing, etc. At the end of the day it means that the second half of the infinity loop of the customer’s journey–the usage and owner experience half–\will require as much skill and focus as marketing has traditionally reserved for the consideration and selection first half of the journey.

### Influencers are influenced

Transformed marketers will ensure that ***the customer hears of a company’s value propositions not just from the company, but from influencers as well***. The appeal of a value proposition has never been limited to just differentiated *claims*; it has also been enhanced by differentiated *communicators* of those claims–think of celebrity endorsers. Transformed marketing will work to systematically influence the messages that those influencers provide.

In a digital distribution world, ***digital influencers are replacing the expert advisory role that traditional distributors provided as part of their service.*** Modern value propositions might be delivered most persuasively by influencers who organically surround the buyer, separate from company-sponsored campaigns. Influencer advice used to be bundled with a distribution service model; now that distribution is going direct, independent digital influencer voices are emerging, sometimes through media-based business models. It’s up to marketing to get to know them intimately.

Thanks to the digital datasphere, companies can begin to tackle the question of who influences whom. Those firms that think their customers aren’t part of an influence cascade probably haven’t thought long enough or hard enough about the question. While in some markets the influencer concept is leading to opaque or corrupted influencer networks, in most markets the question is more about deploying resources to win over influencers. A growth area in transformed marketing will be to better map complex influence chain dynamics with increasing clarity, and by doing so learn to invest in, if not formally manage, the influencers that deliver best results.

**Storbacka**: This comes across as a major shift – basically challenging many of the foundations for marketing. This must have multiple implications for how marketing works in the future.

**Moser**: Yes, exactly! But there is more. Digital does not just transform the value propositions that a company markets. It also transforms the processes by which marketing is done.

## Transformed processes

Marketing leaders will guide the transformation of the core marketing process from digital as alternative marketing to digital as default marketing. To succeed, this process shift must also shift culture: from one of long-prepared broadcast message delivery to one that seeks to listen and understand the customer before responding in a personal manner, drawing on intelligent content that is supported by dynamic segmentation, and equipping marketing to engage in an individualized relationship and journey with each prospect and customer. This newfound capability will enable marketing to travel further with the customer into territory traditionally owned by sales (which may in parallel be working its way into marketing’s territory through its own digital transformation). The resulting mandate will call for effective trust building, bridge process development, and data integration between the marketing and sales functions.

What emerges will be a new core marketing process–let me provide more detail.

### Digital becomes default

Until recently, digital has been the alternative way to market: there was mainstream marketing and digital marketing. ***What’s new is that digital is becoming the default way to market.*** We are on the verge of digital marketing becoming just plain marketing, with other marketing becoming the exception.

This is already visible: CMO surveys show that more than 50% of all marketing spend, from ads to headcount to technology, is now focused on digital channels. It’s driven by customers, whose time spent on digital media properties has only grown, and whose behavioral demographics indicate it will only grow further. Only the inertia of marketing’s past will for a few more years keep the ‘digital’ modifier in front of marketing–it’s no longer needed.

The biggest shock delivered by the emergence of digital-as-default marketing is not a technical shock, although we all know there is a universe of new concepts, technologies, and techniques to learn. The biggest shock is a shock of very human proportions. ***Marketers need, at a human level, to shift from being one-way communicators, who are obsessed with their message, to two-way relationship builders who are obsessed with customer dialog, including listening hard to interpret what the customer is trying to say.***

It’s important to be clear about what new capabilities digital is and isn’t giving marketers. Digital is not giving marketers a mouthpiece or a forum to speak. Marketers have had a mouthpiece since the printing press and have been using it to put advertising in newspapers (since 1704) and magazines (1853) for centuries. Marketers were given an even more powerful mouthpiece through telecommunications and have been using advertising through radio (1922) and TV (1941) for decades.

While marketers have always had a mouth and a public square to speak in, digital technology is giving marketers ears to listen, a mind to process what it hears, the ability to say something in return, and a private room in which to carry out customer dialog. It’s equipping the marketer for conversation. The inbound customer might speak first; marketing needs to listen hard to understand why the customer began speaking before saying something back. The outbound company might speak first; marketing needs to listen carefully to understand what part of its message was relevant and worth speaking further about.

This frames why the biggest process transformation challenge of digital-as-default marketing is a cultural challenge. Now, in the blink of an eye–counting in marketing years–marketing is being called upon to become good at listening, good at clarifying what the customer meant to say, and good at building an authentic human relationship in the intimacy of a private room.

### Content is the new bottleneck

This sets up the second marketing process transformation challenge. When digital becomes the default process for marketing, ***content becomes the new bottleneck*** to marketing impact. This is because creative content teams are set up to prepare the carefully planned one-way speeches that marketers have historically been delivering. Now the creative content team needs to support marketing’s new capability to have a private room customer dialog, where every word that marketing says depends on what it heard, and how it interpreted what its dialog partner–the customer–wants to hear.

When technically implemented, digital marketing provides a request to the content team using ‘if/then’ algorithms that supports a simple line of reasoning: “If this is the customer who I think it is, and if they’re trying to accomplish what I think they’re trying to accomplish, then here’s what content would further develop our relationship. I need to share this content in this channel format with this message in order to move our relational and commercial journey forward”.

The reason content becomes the new bottleneck is that it is a lot easier for digital marketers to learn to program if/then statements, which explode the amount of customized content that is needed, than it is for the marketing content department to produce all that content. The more sophisticated the if/then rules get, the greater the bottleneck becomes. How can the creative content team produce enough of the right content to support the aspirational customer relationship development represented by those if/then rules? At the same time, how can marketers spot declining return on customization to know when to stop?

The answer is intelligent content. Intelligently structured and intelligently applied, modularly built and machine-learning assembled content can provide the content agility that is required by the demands of private-room customer dialog. ***With intelligent content, marketing can carry out its transformed process of building strong customer relationships rooted in dialog and understanding, one customer at a time.***

This is easier said than done. Content departments need to rethink their mission, core processes, technology platforms, and skillsets. They need to ask themselves, ‘how do we reimagine content packages for a digital world?”; and “How do we use automated intelligence to reshape content modules into unique combinations, allowing ML/AI to begin to create the content itself?”

Companies in regulated industries (healthcare, financial services) have an additional roadblock: “How do we gain regulatory approval of modular content blocks rather than as monolithic outputs, and gain pre-approval for appropriate combinations of modular content?”

### Segments are redesigned

A key enabler of digital-default marketing and agile content is a third transformed process–***the development and application of dynamic segmentation schemes***. In an analogue world, companies and their marketing teams needed company-wide agreement on a segmentation scheme; “our four personas” or “our eight segments”. Everything that marketing and product management did ideally conformed with that segmentation.

This was a critical and positive marketing impact driver. It enabled marketing to move beyond communicating the company’s message in the middle of the public square to the entire public audience, and instead enabled marketing to divide the audience into groups, sending each group to a corner of the public square for delivery of more targeted messages. Dynamic segmentation takes this one step further to pose a challenging question: “How can changes in the process of segmentation help marketers evolve from speaking one-way to groups in the corner of the public square to carrying out private room dialogs with one customer at a time?”

The technical answer is to unpack the multi-factorial analysis that produced a company’s standard segmentation scheme in the first place. Behind every quality traditional segmentation scheme lies some form of cluster and factor analysis that incorporates multiple customer dimensions. Despite the valuable contribution these schemes have played historically, going forward they can stand in the way of the more granular and dynamic segmentation needed to deliver private customer dialog.

What digital makes possible is to store single-factor segmentation dimensions in a purer and simpler form, and then combine them later when a specific customer segmentation is needed. This implies ***a larger number of more narrow segmentation schemes available in a system that can be combined throughout the customer journey to serve as the best segmentation expression at a specific point in time, for a specific type of customer.***

Here is a helpful analogy drawn from an unlikely place–your local bar. If you want to order a whiskey, your first decision is to choose either a blended whisky–made by a master blender in the factory lab who has pre-blended multiple varieties to achieve a dependable, enjoyable taste–or choose from a range of single malt whiskeys, each providing a distinct territorial expression. Imagine that the master blender now stops by the bar and offers you a third choice: line up several single malts across the table and, given the taste experience you are seeking that night, he would micro-blend a unique combination to match the moment. Multi-factorial segments are blended whiskeys; dynamic segmentation schemes are the blend-for-the-moment third choice.

A key success factor in implementing any segmentation scheme digitally is marketing’s ability to reliably recognize and assign segmentation values for a specific customer. There are two challenges worth mentioning. One is that early in the relationship development process the customer is often not well known. Deciding on the optimal way to speak to a customer who is not known well enough to be segmented can be tricky. One leading security software company whose product came pre-bundled with new computers found that a large percentage of new computer owners de-installed its software within hours of turning their machines on. When studying how to curb this unwanted behavior, it found two very different customer segments: sophisticated IT users who were offended that the PC company was trying to make a security software choice for them, and unsophisticated IT users who didn’t think they needed security software at all. It had strong but different arguments to make to each group but that hinged on its ability to recognize and classify a customer accurately into the right segment. In the meantime, and to literally buy time, what should be said to the unknown customer based on probabilities?

A second challenge arises from the fact that only some of the customer characteristics that matter in dynamic segmentation are permanent. Some temporary dimensions involve what stage of a pre-purchase journey the customer is in, with what level of engagement. Other temporary dimensions involve where the customer is in a given workflow or lifeflow, assuming that marketing has visibility into the ownership stage of the lifecycle.

The above overview highlights the three key aspects of dynamic segmentation. First, the blend of segmentation schemes that are deployed at a given moment in the customer’s 360 journey will change. Second, some of permanent customer characteristics will change from unknown to known or from wrongly guessed to correctly confirmed as customer knowledge rises. Finally, temporary customer characteristics that capture contextual customer states will fluctuate. The result is a much more powerful approach to segmentation than the monolithic segmentation of the past.

Despite its challenges, a change in the process of segmentation can be a significant enabler of overall marketing impact. A wide group of stakeholders both in and out of marketing need to get together, agree on the business opportunity logic behind new segmentation, and then align on dynamic segmentation architecture.

### Marketing starts to sell

The last transformed process, already implied earlier in the interview, is that ***marketing-sales bridge processes must be built.*** In a digital-default marketing world, both marketing and sales may know the same customer, have siloed data on the same customer, and have different tactics in mind for that same customer. The starting point is usually a messy one, requiring basic hygiene. Sales and marketing might have a CRM record in Salesforce.com, and a different record in Marketo. If customer names are listed differently or if they’re not the same customer ID, progress starts by fixing the basics. If notes from the sales call didn’t get back to Marketo, or if information about what the customer did on the website is not known to the sales force, then there’s a next-level problem to solve.

Once mechanics are fixed, the real upside work begins. Regular marketing reports should highlight accounts that deserve sales prioritization; self-service portals should exist for sellers showing which topics and marketing assets are being reviewed by which prospective buyers.

Most important, ***marketing and sales will need to be able to speak to the customer with one coordinated voice.*** This will become particularly important as digital marketing extends into traditionally selling territory, and as digital selling teams similarly move upstream into what was regarded as marketing’s turf. Only by working together can the process of moving an individualized customer relationship through the sales funnel come to a satisfying ending.

**Storbacka**: Am I right to suggest that the process changes that you describe call for marketing to step out of its organizational silo, or at least work more closely with many other functions?

**Moser**: At a minimum, marketing needs to integrate in new ways and negotiate collaborative governance at new handshake points with sales and with post-purchase customer success. But there are additional types of silo spanning to consider. This is what my third transformation category covers. The digital ripple effect on marketing, described so far as transformed propositions delivered through transformed processes, also demands and enables several types of transformed partnerships.

## Transformed partnerships

Marketing leaders need to forge new partnerships with stakeholders both outside and inside the walls of corporate marketing. First, marketing needs to create two-way sharing of customer journey insights not only with sales, but also with its priority channel partner marketers, making best-practice data sharing a new condition for top-tier partnership. Simultaneously, corporate needs to redefine the kind of value-added that it expects of geographic marketing as traditional sources of value are falling and new sources of value rise. Within the marketing team, performance management software tools are enabling leaders to more effectively partner to shift resource allocation logic away from siloed activity levels, toward integrated strategic objectives that better align with business growth strategy and impact. Finally, marketing analytics leaders must pursue embed-and-extend partnerships with a wide range of marketing stakeholders to create a pervasive performance culture.

### Firms and channels share insight

The first partnership transformation centers on why ***marketing should incorporate two-way data sharing into the design of its channel partner programs***. Channel partners may be physical and e-commerce retailers in B2C markets; they may be brokers, resellers, e-commerce, and value-added resellers, service providers, systems integrators, or independent software vendors in B2B markets.

Corporate marketing teams are using digital marketing to reach past channel intermediaries and establish increasingly direct end-consumer relationships. Some end-customers are responding, creating a three-way company/channel/customer data flow. This can raise practical problems. Both company marketing and channel partner marketing can end up with the data equivalent of one hand over one eye. Each will see one part of the customer’s journey, one portion of the customer’s activities, and will as a result draw an incomplete or inaccurate view of how the customer is behaving, leading them to make less than best moves in advancing the customer’s relational and commercial journey. ***Digital marketing will be most successful if company marketers and its priority channel partner marketers gain full visibility by sharing end-customer insights***.

This is similar to how digital marketing shares end-customer relationships with the sales force, but with more constraints given legal and competitive complexity. Some of the same ‘fix-the-basics’ to-do list that we prescribed for marketing-sales bridge processes applies to channel partners as well. Where channel partnerships are unique is in the design of channel partner programs. Traditional channel programs exist in a tiered manner–platinum, gold, silver–based on company dollar volume sold by the partner, with potential added incentives around co-op advertising levels, rank-order in channel partner recommendations, and achievement of certain product mix targets.

Transformed marketing adds a new partnership angle: intensive data sharing as a new condition for top-tier partnership, and basic data sharing for any level of partnership. Whether that literally takes the form of data or of cookie sharing–again compliant with data regulations–the core principle is to enable the customer to experience seamless coordination while improving channel partner and company results thanks to shared visibility.

Whether a company can execute on this transformation depends on the attitude and strength of its channel partners. If partners are opposed to data partnerships and are stronger in the value chain than the company they distribute, then not much will happen. However, if key channel partners are like minded or are significantly weaker than the company they distribute, real change can occur.

### Geographic value-added shifts

Channel relationships are adjacent to a second key partnership that is being transformed–that between corporate headquarters marketing and geographically-based marketing. Because headquarters is a funding ‘hub’ and geographies are recipient ‘spokes’, geographies constantly carry the burden of proof for their value added.

The value of geographic marketing in a digital era is more a story of “shift” than one of rise or fall. Thanks to digital, ***the value-added of geographic marketing to headquarters is evolving, with traditional sources of value falling and new sources of value rising.***

Digital is directly or indirectly *hollowing out traditional sources of local marketing value added*. Language translation software is increasingly accurate, lowering local language value added. Product homogenization, a result of income segment convergence–meaning that we see more middle class in developing countries, more polarization in rich countries–has helped standardize product lines, lowering product diversity value added. The emergence of a global customer culture in some parts of the market lessens the value of local team insight. Finally, the rising capabilities of remote teams via videoconferencing is lessening the need for many feet on the ground.

At the same time, digital is directly or indirectly *creating new sources of local marketing value added*. The emergence of mega markets rivalling the US, such as China, the EU and India, may justify more dedicated investment. The proliferation of micro-cultures, developed in parallel to–and in response to–global culture, re-introduce a new form of local insight value. The rise of Account Based Marketing (ABM) draws on local data sources and regulations plus decision maker insight sourced from local sales teams. And finally, the necessity to digitally collaborate more closely with channel partners, leads marketing to need talent in the field.

The challenge and opportunity for geographic managers is to ***use technology to diminish costs where digital erodes employee value added, then invest in new skills that create new value***.

### Strategy and allocation unite

The final two transformed partnerships are inter-related, as were the first two. These next two partnerships address digital’s impact on marketing resource allocation.

Digital technology–in the form of Marketing Performance Management (MPM) software–is enabling marketers to shift their approach to Return on Marketing Investment (ROMI) resource allocation and measurement. This shift is welcome and needed, because it moves marketing leaders away from an often-dangerous marketing- activity ROMI approach toward a healthier growth strategy–marketing objectives–marketing activities ROMI approach. In doing so, ***modern MPM creates the possibility of a transformed partnership among marketing leaders to allocate marketing resources for optimal business impact.***

The origins of MPM trace to advertising, where marketers first spent large sums. It then expanded to other marketing activities, an intuitive extension as each activity mapped to an accounting line item budget. One can argue that this turn of events did more harm than good. Given that some activities had short-term, quantifiable benefits, while other activities had long-term, hard-to-quantify benefits, this activity-based ROMI approach led marketers to an imbalanced focus on unit volume acceleration through short-term incentives while failing to maintain high equity assets that drive a differentiation premium.

This triggered an important analytical response–the treatment of brand as a financial asset with tangible balance sheet value through a more sophisticated, quantitative and time-lagged model that gave brand managers a voice in ROMI debates. These models remain critical for supporting high-level “brand v. demand” allocation considerations; however, they don’t substitute for operational marketing resource optimization.

Enter MPM software. ***Marketing leadership teams can use MPM tools - supplemented by program portfolio governance, program design and funding processes, outcomes analytics, and agile program management – to partner together for business impact ROMI.***

MPM tools are still immature, having been originally designed for narrower use. Nonetheless they can enable marketing to plan and track at a unit of investment–the Integrated Marketing Program (IMP)–that directly supports a company growth goal. Each IMP branches into marketing objectives, and each objective branches further to multiple marketing activities. Through tagging, MPM tools become a large ‘pivot table’ converting IMP objectives into multi-activity game plans with draws on accounting budgets. During planning MPM becomes a real-time iterative translator between strategy, activity, and finance.

An MPM information structure can enable resource allocation based on efficacy in achieving business goals and marketing objectives, using outcome oriented KPIs. Management can vet and improve the quality and fit of a team’s marketing strategy and tactics in addition to the basket of activities that they choose. Activity resourcing can be optimized in context, informed by that activity’s effectiveness in meeting the specific company business goal.

### Analytics embeds and extends

This final partnership transformation discussion will share some parallels with the resource allocation partnership just discussed. ***A transformed marketing function will require multiple forms of analytical support, addressing a wide range of structured to unstructured issues, in order to bring about widespread use of analytics.***

Marketing analytics was born in an advertising and brand building era. In the past decade, its scope has exploded in sync with marketing’s growing demand generation role and the astronomical growth of customer and marketing data. Most analytics teams are stretched for resources, with a backlog of varying requests that make support a challenge to prioritize.

In this context ***marketing analytics can pursue an embed-and-extend partnership agenda with a wide range of marketing stakeholders to support pervasive ROMI capabilities.*** It’s helpful for the analytics team to structure analytics support around three delivery formats. Each format requires a different type of partnership with different marketing stakeholders for effectiveness.

The first support format is to partner with tool users, with a goal of empowering them to do their own analytics using functionality embedded in their platforms. Every martech tool has embedded analytics. Most focus on measuring returns on that platform’s data set, tied to its activities. Such a perspective is helpful to tool users but not relevant to the outcomes-oriented questions of senior marketing executives. The support goal of the analytics team should be to highly leverage its time supporting these stakeholders–user tool groups is a good example–in order to free time for less-structured, higher-value analytics support.

The second support format helps each Integrated Marketing Program (IMP) leader to structure, implement, and apply the right analytics framework for their particular IMP. This will require a custom approach per IMP, likely using a data lake; over time patterns will emerge where similar IMPs can benefit from similar analytical approaches. One key to success is to structure analytics for each key IMP objective, not just the overall IMP, so that key program elements can be evaluated instead of delivering all-or-nothing IMP assessments.

The third support format is the least structured. It involves partnering with the marketing leadership team to structure and answer an ongoing series of hypotheses and questions that leadership would like to explore to improve marketing’s effectiveness. Keys to success include maintaining a pipeline of valuable questions, prioritizing those questions continuously, and bringing back answers that are action oriented.

How much of a transformation this represents for analytics will depend on each company’s starting point. Transformation may involve a shift from brand to demand analytics. It may involve a shift from light, reactive support to a systematic and strategic support. A team whose support is structured in the way we’ve discussed will enable the use of analytics to become a pervasive and informative force throughout marketing.

## How do you make the transformation happen?

**Storbacka:** The transformations that you describe are comprehensive, complicated and challenging, and are likely to take quite a long time to implement. Even contemplating the implementation of the changes will make any marketing executive exhausted. How companies are approaching this challenge?

**Moser**: Focus is key. Leaders should choose the few trends that matter to them most, not tackle them all. Given that principle, we see two types of change happening, CMO-led and CEO-led.

When the CMO leads change, he or she typically develops a proactive marketing transformation agenda that sets out a vision for how to modernize the marketing function. The agenda is unique to each company, consisting of three to six impact areas over several years where “from…to” transformation is critical. These multiple changes ladder up to a coherent theme that the CMO develops, articulating a value proposition for the future role of marketing in the company. Armed with this vision, the CMO asks the CEO/C-Suite for transformation funding, because most marketing organizations are funded to carry business-as-usual operations, not to reinvent. The transformation typically lasts two to three years, with annual accountability for what transformation has been accomplished and what business impact has been produced.

I’ve found that most CMO’s have the desire to do this. The only question is whether that desire rises from a “do it someday” to a “do it now.” We see certain patterns in the triggers that compel CMOs to act: new-in-role CMOs may believe that starting strong is key to making their mark; CMOs may want to prepare a game plan ahead of the arrival of a new CEO; CMOs may decide that one year’s annual plan will be transformation vs. incremental; an enterprise-wide digital transformation initiative may reach marketing; and/or marketing may plan for a shift concurrent with a major business shift (often M&A).

A CMO-led marketing transformation agenda is the best opportunity for marketing to step forward in playing a more strategic role within the company. CMO’s have a markedly different chance to frame the discussion if they have led the thinking. That’s why it’s good to act with urgency, because there is another scenario for transformation that is less promising–a CEO or COO-led transformation.

The CEO may approach the transformation from one of several angles. The CEO often sponsors enterprise-wide digital transformation, including marketing. In that scenario marketing will be competing with other functions making similar transformation requests, often with a cost cutting promise that attracts capital more easily than marketing’s promise of better growth.

In a second scenario the CEO may want to build direct-to-consumer business. In this case the CEO may either expand marketing’s role or may shrink it–in the latter case bringing in a new Chief Revenue Officer or Chief Monetization Officer and cutting marketing out.

In a third scenario, the CEO makes a judgment call about where the marketing function ends, and the sales function begins–especially if there is tension between the two. There are two “grey zone” areas that get a lot of attention, the first being: who does digital nurture in the marketing qualified lead (MQL)–sales qualified lead (SQL)–proposal stages. The second area relates to who handles inside sales/remote sales, which at first glance appears to be a sales function, but which is increasingly being supported by best-move decision rules and content rooted in digital marketing infrastructure. The CEO may also create an Executive Vice President of Marketing and Sales role and make that executive responsible for working out the grey zone as it shifts between sales and marketing over time.

In the final scenario, the CEO appoints a new executive to do one or more things that a modern marketing function might do, but isn’t judged as capable of leading. The most common responsibility carve-outs are around data (Chief Data Officer), digital (Chief Digital Officer), experience (Chief Experience Officer) or customer (Chief Customer Officer). In each carve-out the role of marketing shrinks somewhat.

You’ll notice that there is a massive choice point here. Either the CMO proactively creates a transformation agenda to move the company forward, and through that agenda moves marketing’s role forward within the company–or the CEO/COO proactively creates a transformation agenda and typically reduces marketing’s role in the company’s future. This is why it is critical for CMOs to get ahead of the Transformation of Marketing.

## Implications for marketing scholarship

The multi-faceted transformation that Ted Moser is talking about points to numerous needs and opportunities for marketing scholarship to change–both when it comes to research and teaching. One key question is whether universities are preparing our students for this “new normal” by properly educating them.

For academic research to provide insight into the transformation–and the related capability and management issues – research should focus on the less mature areas of the change. This may be counter-intuitive for many scholars, considering the risk averse journal review processes that regularly stamp out innovation (Moorman et al. 2019). But at the same time, I believe that the time is right for more bold explorations of the boundaries of marketing.

There are obvious needs to understand more about how the new digital tools can be integrated into marketing processes. In addition to these, I would like to highlight three both academically and managerially important topics. First, taking work- and lifeflows as a starting point for understanding value creation changes many dominant mainstream marketing perspectives. It is important, however, to understand that these flows are not the same as “customer journeys”. They entail several customer-journey parts (with many different providers) but the flows are larger and much more complex (c.f. Hamilton and Price 2019). As digitalization makes resources more liquid, organizations and individuals have access to an extensive variety of market and nonmarket resources that they can integrate into their work- and lifeflows. The key to these flows is to understand what the organizations or individuals are trying to achieve–what are their goals? A provider that helps them achieve their goals is involved in value creation, whereas providers that make it more difficult for them to achieve goals essentially destroy value. In marketing scholarly language this relates to a need for more research related to the formation of “use-value” or “value-in-context” (Vargo and Lusch 2017), and research on what marketing strategy and marketing management processes look like, if they take their starting point in the work- and lifeflows.

Second, and as a consequence of the above, marketing needs to look beyond the seller–buyer dyad, to see the dyad as part of a larger network or system of stakeholders who contribute to the creation of value (Hillebrand et al. 2015; Nenonen et al. 2019). This systemic view implies that the locus of value creation moves beyond the borders of the firm, i.e., the value emerging in the work- and lifeflows is co-created with a multitude of stakeholders, not only by the firm and for the customer (Tantalo and Priem 2016). Therefore, marketing needs to free itself from the mainstream “firm-based and value-capture-centric view” and move towards a “system-based and value-creation-centric view” (Amit and Han 2017). In a systemic view, revenue development alone cannot be used to measure the success of marketing and sales. Hence, to support future marketing management new measurements are needed.

Finally, in this systemic worldview, there are several important issues related to work divisions and boundaries, both between functions inside organizations and between various stakeholders in the marketplace. Working together with influencers and channel partners to better support the work- and lifeflows of customers entails many challenging and interesting research questions related both to the sharing of data and to the sharing of successes (and failures). And, as noted in the interview, marketing and sales are becoming more integrated–a development that has been accelerated by the COVID-19 developments. Further research is needed to better understand how an integrated marketing and sales function should operate, and what new capabilities firms need to develop.
